# Virus Inactivation by Catalytic Air Converter Filters: The Role of Coating Methods

**DOI:** 10.1021/acsomega.5c05992

**Published:** 2025-08-28

**Authors:** Alicia Gómez-López, Ana Serrano-Lotina, Ángela Vázquez-Calvo, Nicolás Coca-López, Paula Llanos, Teresa García-Castey, Antonio Alcamí, Miguel A. Bañares

**Affiliations:** † Spectroscopy and Industrial Catalysis Group, 16379 Instituto de Catálisis y Petroleoquímica (ICP), CSIC, C/Marie Curie 2, Madrid 28049, Spain; ‡ Immunity and Viromics Group, 70698 Centro de Biología Molecular Severo Ochoa (CBMSO), CSIC, C/Nicolás Cabrera 1, Madrid 28049, Spain; § Escuela de Doctorado, Universidad Autónoma de Madrid, Madrid 28049, Spain

## Abstract

The airborne transmission of pathogens presents a high risk of infection in closed environments, where we spend more than 85% of our time. These infectious respiratory particles contaminated with pathogens remain infectious for hours and can be transported over long distances. To mitigate this threat, it is necessary to develop technologies that can remediate, remove, or decrease airborne pathogen concentration or infectivity in air. In this work, patented catalytic polymeric converter filters were prepared by spray coating and dip coating. The preparation of spray-coated filters was optimized by evaluating the effect of the air pressure and the distance between the filter and the airbrush. Micrographs and hyperspectral Raman maps demonstrated that the air converter filters prepared by spray coating present a more homogeneous coating than the dip-coated sample. Converter filters prepared by spray coating at 1 bar and a 6 cm airbrush-filter distance showed the best coverage. Moreover, this optimum filter exhibited the best adherence, with a mass loss of 0.5% after 180 min of ultrasound treatment. This catalytic polymeric converter filter has shown a reduction of 4 logarithm units after 60 min of exposure to HCoV-229E at room temperature. Overall, this work introduces an optimized spray-coating method as a low-cost and simple process to prepare a biocidal catalytic polymeric converter filters.

## Graphical abstract

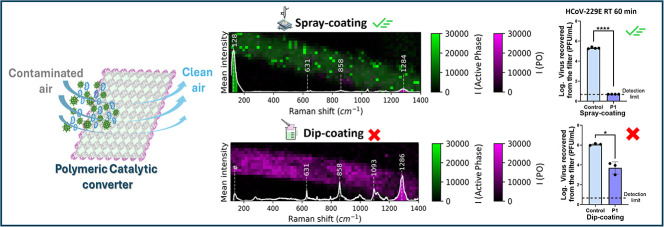

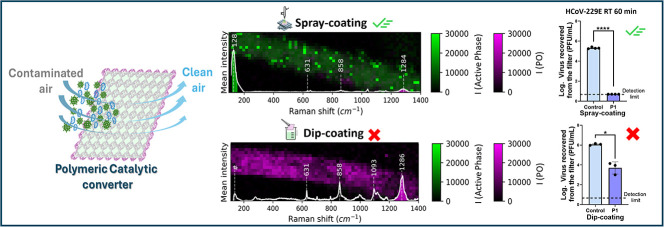

## 1 Introduction

The airborne transmission of pathogens, such as tuberculosis (TB), severe acute respiratory syndrome, or influenza, among others, presents a higher risk of infection in closed environments. In lower respiratory tract infections, *Streptococcus pneumoniae*,*Haemophilus influenzae,* and *Mycobacterium tuberculosis* are among the most frequently encountered pathogens.^1,2^ Pneumonia can range from a mild illness to a life-threatening condition and affects people of all ages. However, it is the leading infectious cause of death in children worldwide. In 2017, pneumonia claimed the lives of over 808,000 children under the age of 5, accounting for 15% of all deaths in this age group. Those at higher risk of developing pneumonia include adults over 65 years old and individuals with existing health conditions.^3^ On the other hand, *H. influenzae* accounts for over 90% of systemic infections. This bacterium mainly causes pneumonia and meningitis in young children and remains a major public health issue in many regions worldwide, with approximately 3 million cases of severe illness reported annually.^4^ In 2023, TB caused 1.25 million deaths. Globally, TB likely regained its position as the leading cause of death from a single infectious agent after three years of being surpassed by COVID-19. In summary, more than 750 million deaths have been reported due to COVID-19 ^5^ and between 290,000 and 650,000 respiratory deaths per year due to seasonal influenza.^6^

The transfer of pathogens can occur through various mechanisms:^2,7–9^

1 Touch, which includes both direct and indirect contact.
2 Spray, which large infectious respiratory particles (IRPs) directly land on exposed mucosal surfaces of a susceptible person.
3 Airborne transmission/inhalation: in this mechanism, smaller IRPs are inhaled by a susceptible person, allowing the pathogen to reach the respiratory tract.

IRP contaminated with pathogens is the main route of transmission of airborne infections, where the lower respiratory tract is the most susceptible.^2^ The probability of infection is much higher in closed spaces with poor ventilation, where we spend more than 85% of our time. These respiratory particles remain infectious for hours and can be transported long distances.^7^

To mitigate this threat, it is necessary to develop technologies that can remediate, remove, or decrease airborne pathogen concentration and/or infectivity in air. Air purification technologies can be classified into physicochemical or chemical aerosolization treatments according to the inactivation mechanism. Within the physicochemical technologies, air filtration is the most widely used.^7,10^ However, pathogens present in IRPs in indoor air accumulate in large quantities in the filters of HVAC systems, where they can multiply, especially if there is high humidity in the filter. In addition, organic or inorganic materials (e.g., dust) retained in the filter contribute to microbial growth. In addition, volatile organic compounds produced by microbial metabolism (MVOCs) can be emitted from contaminated filters.^11–14^

Other air purification methods are based on physical damage produced by ultraviolet (UV) radiation. Yet, UV radiation at high intensities can cause eye and skin damage during practical application.^7,15^ On the other hand, using light combined with oxidation processes (photocatalysis) has been shown to disinfect a wide variety of microbial contaminants using only sunlight or artificial light and a photoactive material. However, the photocatalytic production of intermediates potentially includes organic and inorganic species (such as aromatics, ketones, and alcohols), which may block the photocatalytic reactions and result noxious.^7,16^ The use of plasma damages cell membranes, DNA, and proteins of airborne microorganisms, but it can also release ionic species.^17^ The technology used for years to inactivate airborne pathogens is ozone, which damages the organelles of cell membranes. However, ozone, along with chemical aerosolization or the use of botanical disinfectants—other common technologies used for disinfection, cannot be used in occupied spaces because many of them are harmful to the human organism and corrode electronic devices.^7,18,19^ Thermal treatments can also inactivate airborne pathogens by denaturalizing proteins and deteriorating the cell structure, but it is not widely used because of its high energy consumption, and it cannot be used when people are present.^20^

In this work, we proposed the use of catalytic systems with biocidal properties that can prevent the spread of airborne pathogens by oxidative stress of the pathogen at mild temperatures (25–37 °C). We propose the use of polymeric catalytic converter filters that can be easily used in commercial HVAC systems and that will not need periodic replacements since the pathogens do not typically adsorb on the filter, so the filter does not become saturated. Moreover, no harmful reactive species are released. For the preparation of the catalytic converter filters, we propose the use of polyester as support since polyester is one of the most relevant materials in the textile industry due to its good characteristics, such as high strength, good chemical stability, and poor moisture adsorption. The development of an easy and clean method to improve its weak antibacterial properties is highly desirable in terms of environmental and economic concerns.^21–23^

Several materials based on Ag, ZnO, TiO_2_, and Cu have been used to develop antimicrobial textiles due to their potential antimicrobial activity. Among the metal oxide nanoparticles, those based on zinc oxide present high chemical stability, excellent biocompatibility, widespread availability, cost-effectiveness, and low toxicity. This versatility has made them highly effective in surfaces and coatings, as they can inhibit the bacterial growth and prevent biofilm formation to mitigate the spread of infectious pathogens.^24^ In addition, titanium dioxide is an antiviral material that has been used as a coating agent. However, it presents the limitation of the requirement of UV irradiation for generating hydroxyl radicals and the degradation of the base material.^25^ On the other hand, copper compounds present antibacterial properties comparable to those of other expensive metals such as gold or silver and have been used as antiviral agents because they have a broad spectrum of antiviral activity against both enveloped and nonenveloped viruses.^26^ The American Environmental Protection Agency (EPA) has registered copper as the first and only metal with antimicrobial properties, which kills 99.9% of most pathogens within 2 h contact.^27^ Several studies have reported that copper-based nano- and microparticles exhibit inhibitory effects on microbial and cancer cell growth. Similarly, iodine-based nano- and microparticles have demonstrated effectiveness in suppressing both microbial and tumor growth. Based on this, composite particles combining copper and iodine will produce a synergistic effect, with iodine enhancing the antibacterial activity of copper.^28^ Many authors have reported the inactivation of bacteriophages^29^ and virus as avian influenza virus by copper metal and Cu^2+^ ^30,31^ and the inactivation of human immunodeficiency virus by copper ions and copper oxide.^25^ CuI particles can generate hydroxyl radicals probably derived from Cu^+^, being effectively applied to items such as filters, face masks, protective clothing, and kitchen cloths.^25^

The preparation methods of these catalytic systems include dip-coating and spray-coating of the fibers. Dip-coating is a very simple method that consists of the immersion of the textile in a solution of the biocidal agent. On the other hand, spray-coating is an industrially known method by which solutions or suspensions are deposited by the release of a large amount of submicrometer particles on different types of materials. The spraying method can be easily applied to the preparation of polymer coatings. This technique endows the preparation with uniform coatings in materials with large areas and requires a lower amount of coating solution compared with dip-coating. The spray-coating method exhibits a minimal solution loss compared to dip-coating, where large volumes of coating solution are required to immerse the entire filter, increasing operational costs.^32^ Moreover, it is a simple and scalable process that can be applied to a variety of applications^33–36^ compared to the dip-coating process, which may lead to uneven coverage.^37^ Spray-coating is a faster process because it eliminates the immersion stages required in the dip-coating process.^32^ In contrast, the spray-coating process could present some limitations, such as a part of the sprayed material may not adhere to the substrate, leading to waste and inefficiency; another common limitation is the overspray and rebound losses due to the low-viscosity of the active phase solution. In addition, if the parameters are not properly controlled, there will be variability in spray-angle, distance, and nozzle design, including, in some cases, the droplet coalescence before reaching the substrate.^38,39^ Other limitations reported from the literature include the solvent retention in the coating, affecting performance and safety, and the difficulty adherence to hydrophobic or rough surfaces without pretreatment.^39,40^ The objective of this work is to optimize the fabrication conditions as a low cost, simple, and versatile process for preparing catalytic polymeric air converters. Operation parameters to be optimized were the compressed air pressure and the airbrush-filter distance. Gas pressure directly affects the beam profile and the mean drop size. Optimum parameters were selected as a function of their biocidal capacity, coating homogeneity, and the adherence of the coating.

## 2 Materials and Methods

### 2.1 Materials

Polyester textiles (100% polyester fiber) used in this work were supplied by RS Iberia (Madrid, Spain) with the corresponding certifications (IN 53438, EN 779, and ISO 9073-2). Spray-coating setup combined an airbrush (Badger Vega 2000) with an *X*–*Y* table manufactured ad hoc by “IDC Tecnología de Instalaciones Industriales, S.L”, which allows the automation of the preparation.

Copper­(I) iodide-98% reagent (CuI) was directly purchased from Sigma-Aldrich (St. Lewis, MO, USA), and acetonitrile anhydro ((max. 0.003% H_2_O) ≥ 99.95%, HiPerSolv CHROMANORM Reag. Ph. Eur., Reag. USP, ACS) was purchased by VWR Chemicals.

For the biocidal tests, HuH-7 cells (kindly provided by Isabel Solá and Luis Enjuanes, CNB–CSIC, Madrid, Spain) were grown in Dulbecco’s modified Eagle’s medium (DMEM) containing 2 mM l-glutamine, 100 mg·ml^–1^ streptomycin, and 100 U·mL^–1^ penicillin (complete DMEM) and 5% fetal bovine serum (FBS), incubated at 37 °C and 5% CO_2_. The virus used was human coronavirus 229E (HCoV-229E) (kindly provided by Isabel Solá and Luis Enjuanes, CNB–CSIC, Madrid, Spain). All infectious virus manipulations were performed at biosafety level 2 (BSL2).

### 2.2 Preparation of Catalytic Polymeric Converter Filter

The polymeric catalytic filters were prepared according to the procedure reported in the patent file.^41^ In short, commercial polyester textiles were cut into 6 cm × 6 cm pieces. For the preparation of the spray-coated filters, the system displayed in Figure 1 was used with a constant spray time of 100 s. In this method, different operation parameters such as compressed air pressure and airbrush-filter distance were evaluated. Tested air pressures were set at 0.5, 1, 1.5, and 2 bar, and the airbrush-filter distances were fixed to 2, 4, 6, 8, 10, and 12 cm. In all cases, the final solution volume used for the coating was 12 mL, and the concentration of CuI was 0.1 M CuI (dissolved in acetonitrile). After the spraying process, the spray-coated textiles were dried overnight at room temperature. The dip-coated converter filter was prepared by immersing the textile in a 0.1 M CuI solution. In this method, it is necessary to prepare at least 50 mL of solution to fully immerse the filter (almost five times more than for the spray-coating). After the immersion, the textiles were dried at room temperature overnight. A total of 25 catalytic polymeric converter filters were prepared.

**Figure 1:**
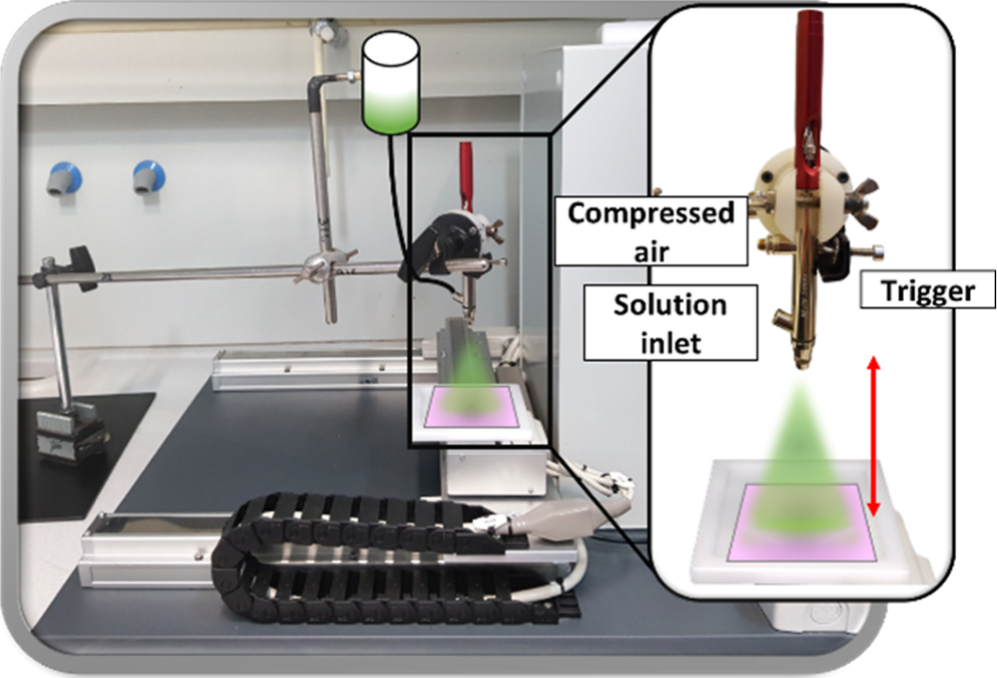
Illustration of the implemented spray-coating system. The active phase is colored in green and the filter is colored in purple.

### 2.3 Filter Characterization

#### 2.3.1 Field-Emission Scanning Electron Microscopy

The homogeneity of filters was determined by field-emission scanning electron microscopy (FE-SEM, Nova Nanosem 230 FEI, Hillsboro, OR, USA; with variable potential 50 V–30 kV). The samples were measured at 6 kV. In addition, elemental chemical mapping has been performed using a new generation energy dispersive spectroscopy (EDS) detector (Genesis XM2i of EDAX Inc.) with a resolution up to 133 eV.

#### 2.3.2 Raman Spectroscopy

Hyperspectral Raman maps were acquired with a Renishaw InVia Qontor confocal Raman microscope with a 20× long-distance objective (NA = 0.4) using a 514 nm laser excitation with 1 s exposure time (around 2000 spectra were collected in each map). The Raman shift axis was calibrated with the help of the main silicon band.^42^ Spikes were removed using an in-house open-source algorithm,^43^ available at GitHub. A linear baseline was fitted to different parts of the spectrum to calculate the area under the different Raman peaks.

#### 2.3.3 Ultrasound Adherence Test

The resistance of the coating to mechanical stress was performed through an ultrasound adherence test at 30, 90, and 180 min with a J.P. SELECTA ultrasonic bath (model 3000865) with a power of 195 W. This method consists of evaluating the mass loss with respect to the initial mass filter, caused by an ultrasonic vibration^44,45^ in a filter placed into an empty beaker glass that is immersed in the bath. The mass loss percentage was calculated with respect to the initial filter mass in eq 1.

1 $$Mass\;loss\;\left(\%\right)=\frac{Initial\;filter\;mass\;\left(g\right)-Final\;filter\;mass\;\left(g\right)}{Initial\;filter\;mass\;\left(g\right)}\cdot 100$$

#### 2.3.4 Virucidal Assays

For the virucidal assay, coated or uncoated (control) textiles were cut into 1 × 1 cm^2^ squares. The samples were sterilized by exposing to UV–C irradiation for 30 min. Hereinafter, 100 μL of viral stock containing ∼10^6^ plaque-forming units (PFU) of HCoV-229E was added above the surface of each polymeric converter filter and incubated for 15 min at room temperature. After incubation, the virus attached to the surface was recovered by washing with 900 μL of complete DMEM containing 2% FBS and centrifuging at 7500 rpm to remove possible residual material. Biocidal activity was assessed by quantifying the number of PFUs recovered from the filter using plaque viral titration assays. Briefly, from the virus solution recovered from the filter, serial dilutions were carried out in base 10 in the virus suspension medium; 200 μL of each dilution was used to infect monolayers of HuH7 cells grown in 12-well plates. The filters were exposed to the virus for 2 h at 37 °C, and after that, the inoculum (the initial substance added to infect the cells) was taken out. Then, the culture medium (DMEM), which includes 0.7% agar, 2% FBS, and 0.09 mg·mL^−1^ DEAE-dextran (a polymer used to enhance virus infection efficiency in cells), was added. Finally, the plates with the samples were incubated for 4 days at 33 °C (temperature used for respiratory virus studies) to allow the infection process. Finally, the cells were fixed with a 2% formaldehyde solution for at least 30 min. After fixation, the semisolid medium was removed, and the plates were stained with 0.02% crystal violet in 10% ethanol and 2% formaldehyde. The plates were washed with water to remove excess crystal violet and allowed to air-dry. Once dried, the resulting PFU’s were counted.

All virucidal assays were performed in quadruplicate. All data are represented as the mean ± standard deviation. Statistical significance of the results was determined by paired *t*-student analysis (GraphPad Prism4) when comparing experimental treatments with regard to the control. One asterisk (*) signifies a *p*-value <0.1; two asterisks (**) signify a *p*-value <0.01; three asterisks (***) signify a *p*-value <0.001, and four asterisks (****) signify a *p*-value <0.0001.

## 3 Results and Discussion

### 3.1 Characterization

#### 3.1.1 FE-SEM Characterization

The active phase dispersion and homogeneity of coated polyester were evaluated by FE-SEM. The micrographs of polymeric catalytic filters and untreated polyester are shown in Figure 2. The untreated polyester (Figure 2b) appears as dark smooth fibers, whereas Figure 2c clearly shows the deposition of CuI on the polyester surface at every pressures and distances evaluated. Those textiles prepared at short distances (2 and 4 cm) presented a non-homogeneous impregnation and agglomeration of the active phase. In addition, the impregnation time was longer since the impregnation cone is smaller, as shown in Figure 2c. In the case of 6 and 8 cm, the surface is more uniformly covered by CuI. Finally, when the airbrush-filter distance increased (10 and 12 cm), the impregnation is again not very consistent. In summary, three preparation alternatives can be considered according to the working distance (see Figure 2a). The wet preparation occurs when the distance between the airbrush and the sample is short, leading to a wet coating that forms an inhomogeneous layer; on the other hand, a dry preparation can be obtained when the sample to airbrush distance is high, as the solvent can evaporate before it reaches the sample, generating a powder coating. Finally, an optimal semi-wet preparation consisting of an intermediate distance achieves a homogeneous layer.

**Figure 2:**
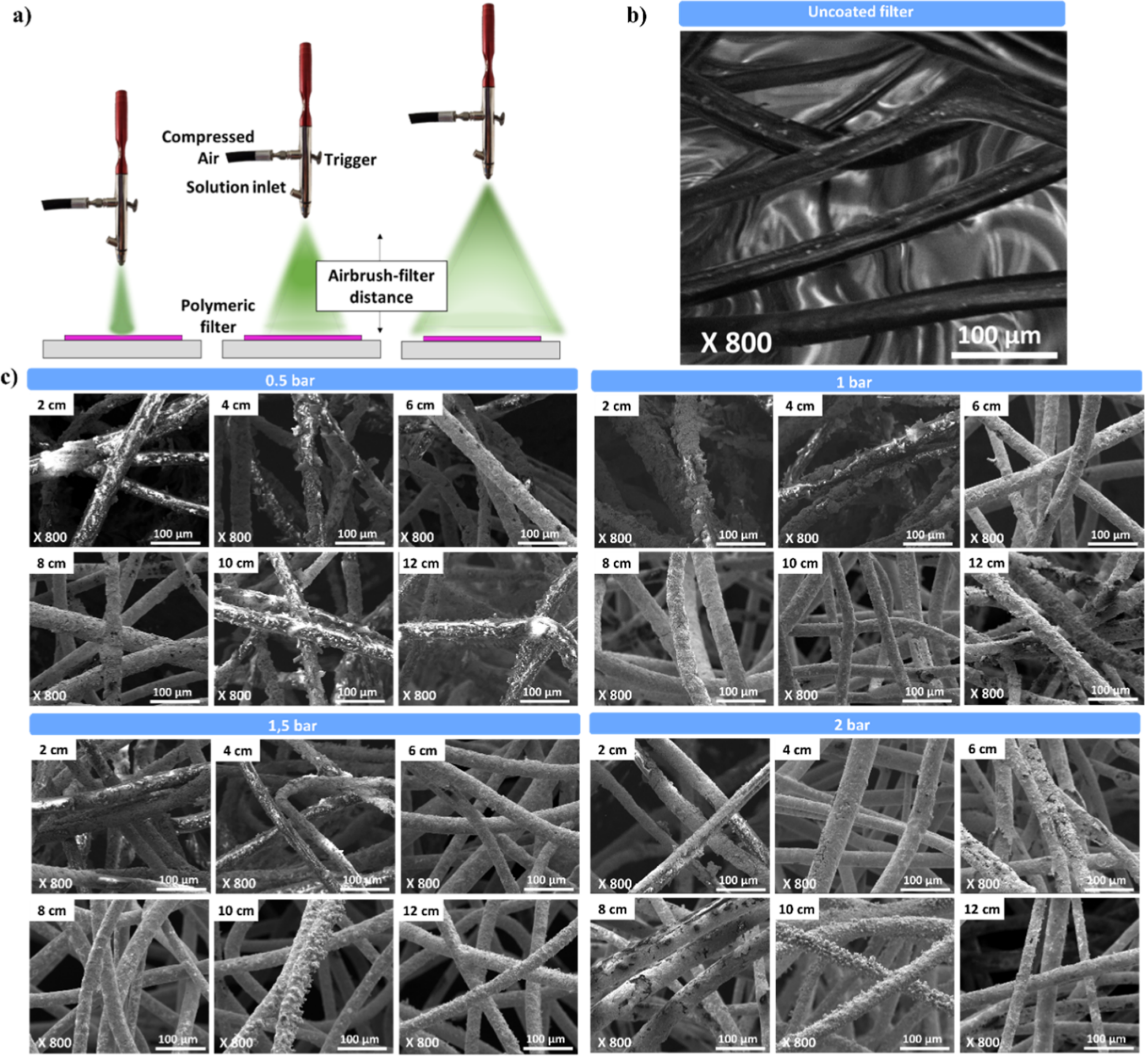
(a) Scheme of spray-coating preparation when changing the distance between the airbrush and the filter, (b) FE-SEM micrograph of uncoated filter (control), and (c) FE-SEM micrographs of the spray-coated textiles at different airbrush-filter distances and pressures.

Regarding compressed air pressure, at low pressures (0.5 bar) fibers are non-homogeneously coated and the agglomeration of the active phase is observed, contrary to intermediate pressures (1 and 1.5 bar) where the coating is more uniform. For high pressures (2 bar), the coverage at short distances improves compared to lower pressures, although the coverage is less uniform and detaches from the polyester fibers compared to that at intermediate pressures.

This study indicates that spray-coated filters prepared at intermediate airbrush-filter distances (6–8 cm) and pressures (1–1.5 bar) exhibited a higher homogeneity of the active phase. Within the optimized conditions, a comparison was made with the sample prepared by dip-coating (direct immersion of the filter in the solution containing the active phase).

Figure 3 shows the micrographs and elemental mapping by EDS of the dip-coated textile and the polyester prepared by spray-coating (at 1 bar and 6 cm). In the former, a poor coating is obtained, where uncovered areas can clearly be observed. In the latter, the polyester fibers show complete coverage. Dip-coating on fibrous materials frequently results in non-uniform coatings due to complex surface topography and variable wettability. The review by Tang and Yan discusses how surface roughness and capillary effects in fibrous substrates can impede the formation of continuous and uniform coatings during the dip-coating process.^46^ For both elements of the active phase, copper and iodine, there is a higher concentration in the spray-coated filter. In spray-coating, atomized droplets are delivered in a controlled manner. This enables better control over thickness and uniformity.^47^

**Figure 3:**
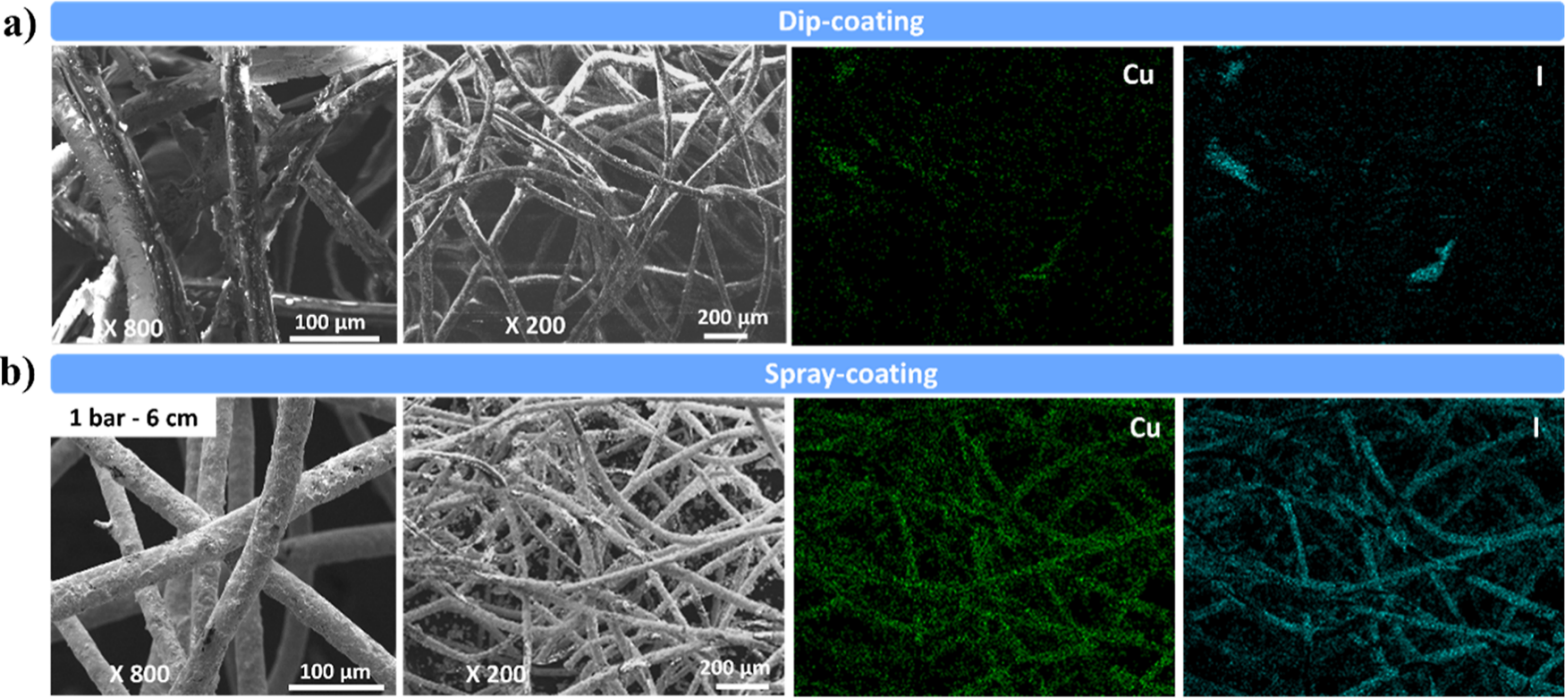
FE-SEM and surface distribution images of copper (green) and iodide (blue) of coated filters prepared by (a) dip-coating and (b) spray-coating (1 bar and 6 cm of airbrush-filter distance).

The drying behavior of spray- and dip-coating processes differs significantly, affecting the uniformity and quality of the resulting coatings. In spray-coating, the fine droplets have a high surface-area-to-volume ratio, allowing for rapid solvent evaporation upon contact with the support. This immediate drying helps preserve the initial distribution of the coating material, reducing the probability of flow-induced defects and promoting a uniform coating. In contrast, dip-coated filters dry as a continuous layer, which is more prone to solvent evaporation gradients and gravitational effects. These factors, as confirmed here, can lead to non-uniform coating. Moreover, dip-coated filters are susceptible to getting saturated and runoff, further compromising coating uniformity.^47^

#### 3.1.2 Raman Spectroscopy

The Raman analyses of uncovered polyester commercial textile are displayed in Figure 4. Figure 4a shows a white light image of the uncovered polyester commercial textile fiber, and Figure 4b shows the white light image of the area inside the white rectangle. The Raman hyperspectral map of this area is plotted in Figure 4c. The mean spectrum of this map is shown in Figure 4d, showing the Raman bands associated with the polyester.

**Figure 4:**
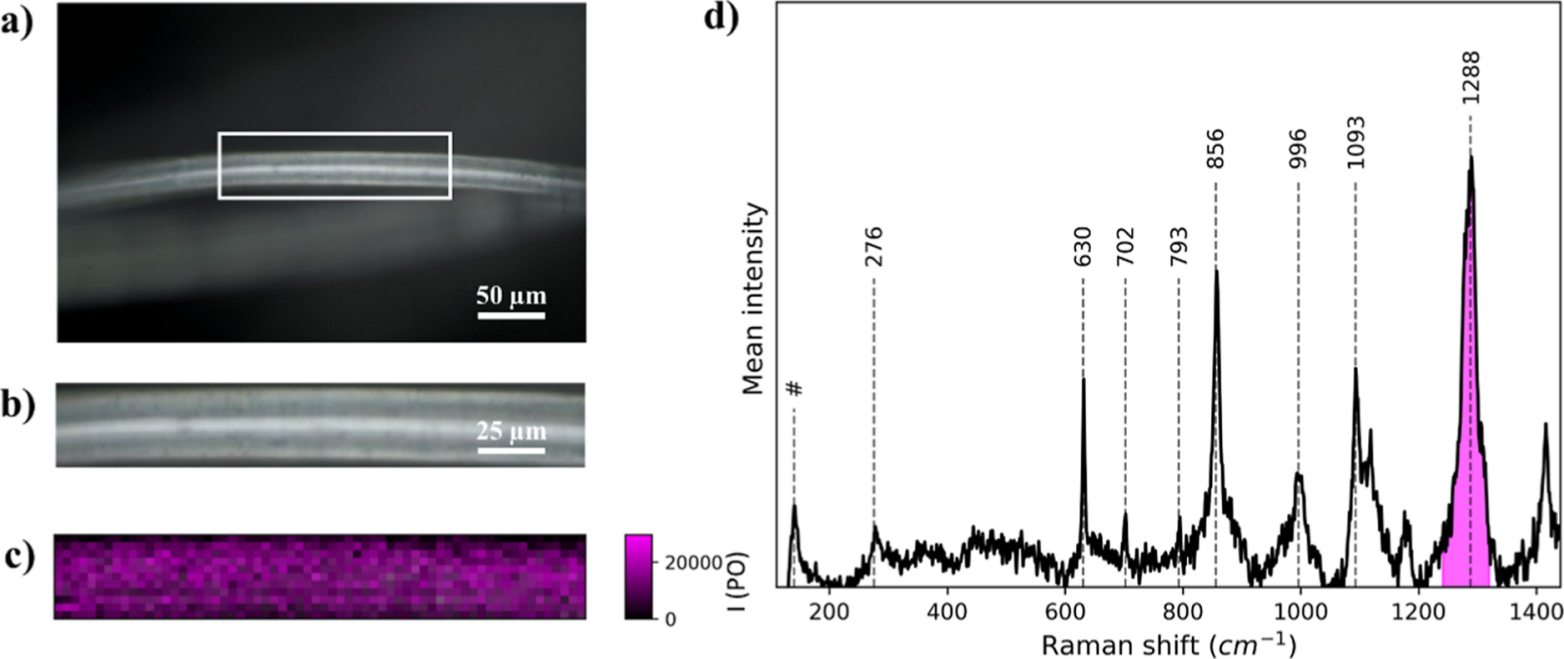
(a) White light image of the uncovered polyester commercial textile. (b) White light image of the white rectangle, (c) Raman intensity map of the area shown in (b). (d) Average Raman spectra of commercial polyester. Corresponds to an artifact due to the edge filter laser cutoff.

The different vibrational bands^48–50^ are shown in Table 1. Briefly, the polyester filter exhibits ring stretching, bending, and deformation in the 200–800 cm^–1^ and 1100–1400 cm^–1^ spectral regions, respectively. Also, the spectrum shows peaks at 1093 cm^–1^ originated by C–C and C–O–C vibration and at 996 cm^–1^ corresponding to C–C stretching. We select the band of 1288 cm^–1^ corresponding to the asymmetric C–O–C stretching vibrational mode, which has the highest intensity in the spectra, to use as a reference for Raman spectral mapping (magenta color).

**Table 1:** Vibrational Bands of Raman Spectra Obtained from Polyester Filter^48–50^

band	vibrational modes
276	C–C stretching (ring), C–C–C bending (ring)
630	ring deformation, d(C–O–C)
702	ring C–C bend
793	C–C–C ring deformation
856	ν_s_(C–O–C)
996	C–C stretching
1093	ν_as_(C–O–C); ν_s_(C–C)
1288	asym C–O–C stretching

Figure 5 shows the CuI Raman spectrum, which presents a sharp peak at 122 cm^–1^; it corresponds to the transverse optic (TO) vibration mode of phonons. Around 140 cm^–1^, there is a small broadening of the peak corresponding to the longitudinal optical (LO) vibration mode. This may be due to the LO peak being submerged in the broad TO peak (Figure 5, inset). The peak widening of the TO vibration mode can be attributed to the presence of the disorder in the structure of CuI.^51–53^ The peak at 122 cm^–1^ was used as a reference for CuI evaluation (green).

**Figure 5:**
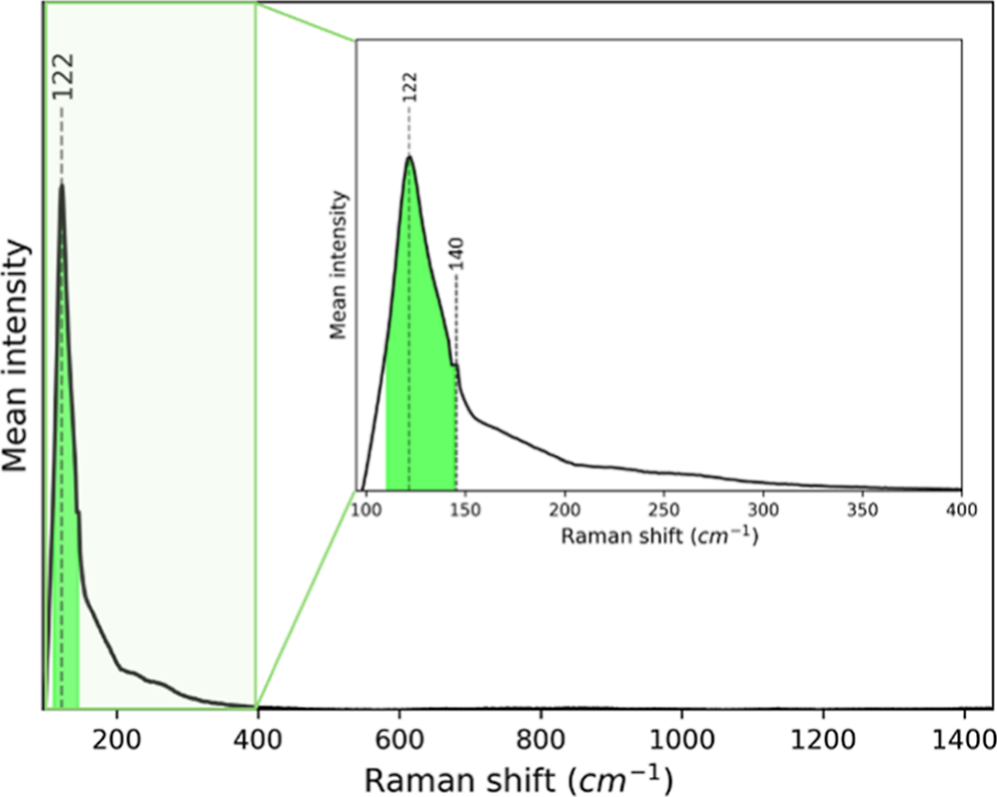
Raman spectrum of CuI reactive agent.

FE-SEM characterization illustrates that the filters prepared at intermediate pressures and distances show better homogeneity and coverage. For that reason, only 5 spray-coated textiles (see Table 2) were selected for Raman characterization with the optimum pressure and different distances and with the optimum distance and different compressed air pressures. For comas also characterized.

**Table 2:** Polymeric Filters Prepared by Spray-Coating Selected (X) for Raman Analysis

airbrush-filter distance	compressed air pressure			
	0.5 bar	1 bar	1.5 bar	2 bar
2 cm		X		
4 cm				
6 cm	X	X		X
8 cm				
10 cm				
12 cm		X		

Figure 6 shows the microscope white light image (Figure 6a), the intensity Raman map associated with the polyester and copper iodide bands (Figure 6b), and the average spectra (Figure 6c). Maps are plotted as a function of the intensity of the 122 and 1288 cm^–1^ peaks corresponding to CuI (green) and polyester (magenta), respectively. The textiles prepared with the optimum distance (6 cm) show complete coverage of the polyester fibers for 0.5 and 1 bar of compressed air pressure. In the intensity maps, the green color is predominant, and in the Raman average spectra, the band corresponding to the TO vibration mode of the copper iodide has higher intensity than the polyester vibrational bands. However, with the same distance but increasing compressed air pressure (2 bar), there are some magenta-colored areas, corresponding to the uncoated zones. We can also see in the average spectra that the band around 1288 cm^–1^ is more intense than at lower pressures.

**Figure 6:**
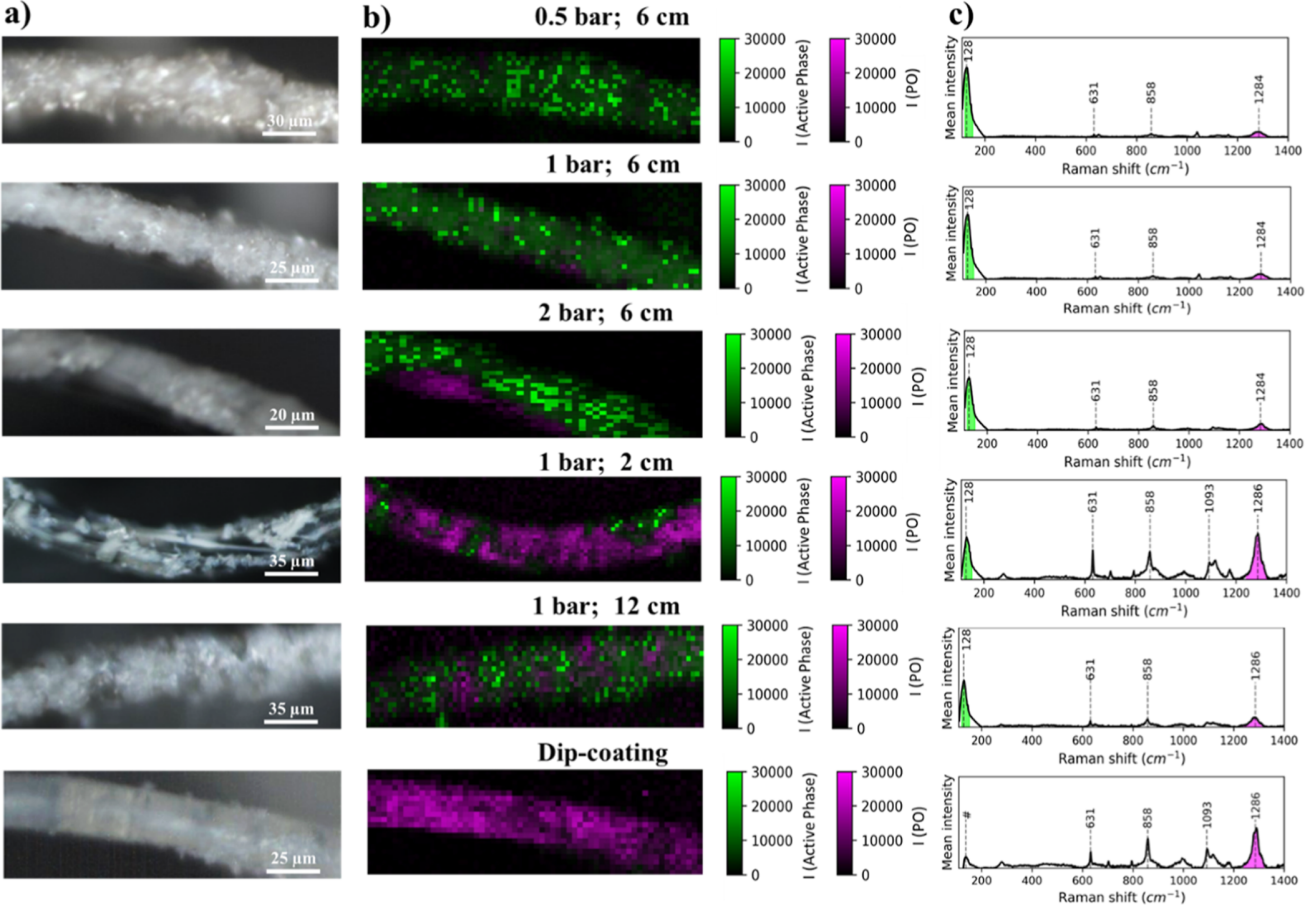
(a) White light images and (b) Raman intensity map of catalytic polymeric filters prepared at 0.5 bar 6 cm; 1 bar 6 cm; 2 bar 6 cm; 1 bar 2 cm; 1 bar 12 cm; and dip-coating. (c) Normalized spectra of catalytic filter air converters at different conditions.

At the optimum spray pressure (1 bar) and at short distances (2 cm), the samples exhibit a less homogeneous coating of the active phase. It is evident that the fibers are clean of CuI. Accordingly, the intensity map exhibited a more extensive area in magenta, corresponding to the polyester phase. Regarding the average Raman spectrum, vibrational bands around 630 cm^–1^ (d­(C–O–C)), 856 cm^–1^ (V­(C–O–C)) and 1288 cm^–1^ (C–C and C–O stretching) are higher than the band associated with CuI. Finally, when airbrush-filter distance increases (12 cm), the coating of polyester deteriorates, detecting some uncovered areas.

When characterizing the dip-coated textile, CuI remains poorly adhered to the polyester fibers compared with the spray-coating method. Thus, in the intensity map, mainly the band corresponding to the polyester was detected.

These results confirm SEM findings, where the optimum airbrush-filter distance was 6 cm, and the optimum pressures were 0.5 and 1 bar.

#### 3.1.3 Ultrasound Adherence Test

To evaluate the resistance of the coating to mechanical stress, the ultrasound adherence test was performed at 30, 90, and 180 min.^44,45^ Figure 7 shows the mass loss of the dip-coated textile and the selection of spray-coated samples, as indicated in Table 2.

**Figure 7:**
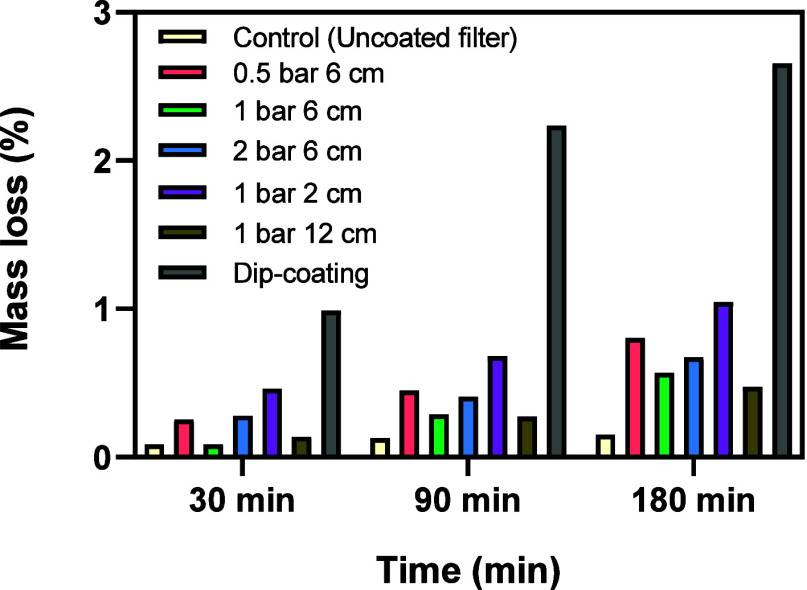
Mass loss (%) in function of the ultrasound treatment time for catalytic polymeric converter filters prepared by dip-coating and spray-coating at different pressures and airbrush-filter distances.

Adhesion in coated systems is governed by mechanisms such as mechanical interlocking, surface energy compatibility between the coating and substrate, and stress development during solvent evaporation. Spray-coating can lead to stronger adhesion by enabling enhanced surface conformity and faster solvent evaporation, thereby reducing the formation of internal stress gradients.^54^ In all of the tested samples, the mass loss increased with time due to the detachment of the active phase. The dip-coated textile is the one that exhibited higher mass loss at all the times tested, reaching a maximum of 2.7% of mass loss after 180 min of ultrasound treatment. This is because, after the dip-coating process, the active phase remains mainly on the edges of the filter, creating an accumulated coating in these areas that easily detaches. Even after 30 min of ultrasound treatment, a mass loss of 1% was detected. For the spray-coated samples, a much lower mass loss was detected, being lower with a compressed air pressure of 1 bar and a distance of 6 cm (0.5% at 180 min). This indicates better adherence of CuI and a higher resistance to mechanical stress. These results support the SEM and Raman spectroscopy characterization findings, demonstrating that spray-coating is the optimum preparation method when conditions of 1 bar and 6 cm were used.

#### 3.1.4 Virucidal Assays

To assess the functionality of the catalytic converter filters, the virucidal activity against HCoV-229E was evaluated by incubating ∼10^6^ PFU on the surface of each textile at room temperature for 15 min. The viral infectivity recovered from the filters was determined by quantifying the number of plaque-forming units. Figure 8 shows the virus recovered from each converter filter and its control.

**Figure 8:**
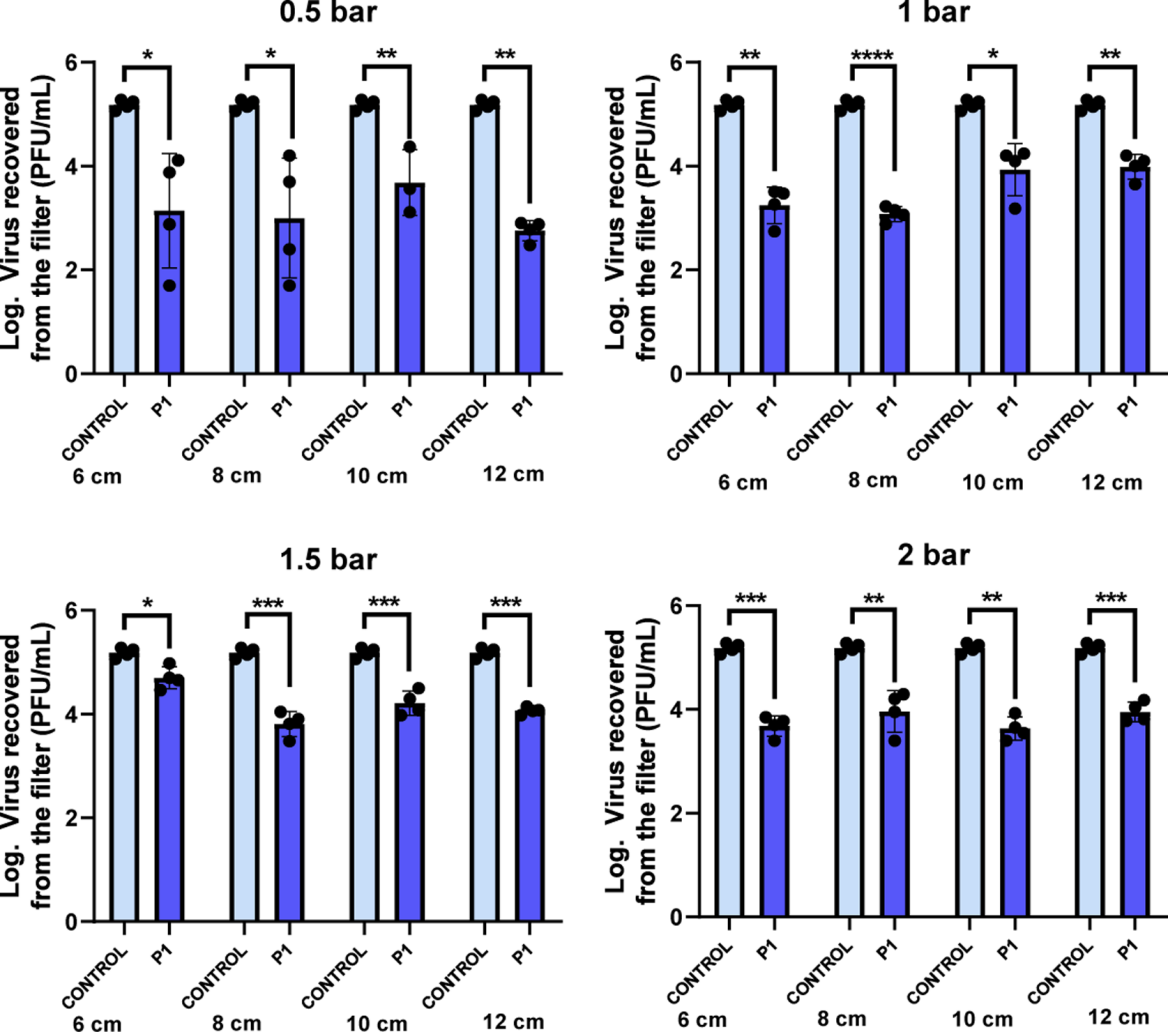
Log of virus recovered after exposure to catalytic polymeric filters to HCoV-229E at room temperature for 15 min. The detection limit of the technique is 5 PFU/mL. *N* = 4; error bars correspond to the standard deviation (±SD). Paired student test analysis was used to compare experimental treatments with control: *p-value <0.05; **p-value <0.01; ***p-value <0.001; and ****p-value <0.0001.

Since the SEM images show a poor dispersion of the active phase on the filters prepared at short distances (2 and 4 cm), they were discarded for these tests. The results show that at 0.5 bar, there is more variability in the results, which can be due to the lower homogeneity of the filter, as was demonstrated in SEM and Raman characterization. However, for 1 bar, replicate assays give more homogeneous results, and for 6 and 8 cm airbrush-filter distances, a reduction of 2 logarithms with respect to the control was obtained. For higher distances (10–12 cm) and higher pressures (1, 5, and 2 bar), results were worse (1 logarithm reduction). In summary, the filters prepared at medium airbrush-filter distances (6 and 8 cm) and at intermediate pressure (1 bar) afforded better performance (reduction of 2 logarithms of virus viability).

The performance of the optimum filter (6 cm and 1 bar) was compared with the dip-coated sample against the HCoV-229E virus at room temperature after 15 and 60 min of exposure (Figure 9). While less than 1 logarithmic reduction was detected after 15 min of exposure when the dip-coated textile was tested, 2 orders of magnitude reductions were observed in the spray-coated material. When an exposure of 60 min was evaluated, the former exhibited 2 orders of magnitude reduction, while the latter afforded, at least, 4 orders of magnitude (results below the detection limit).

**Figure 9:**
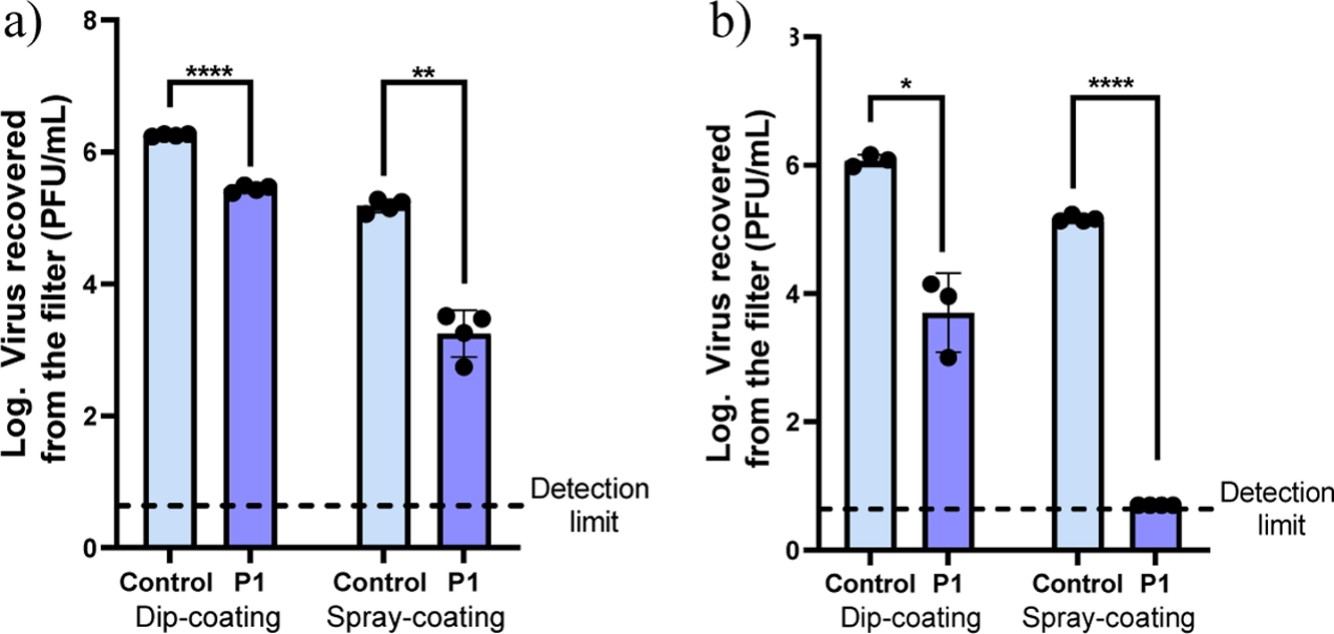
(a) Log of virus recovered after exposure to catalytic polymeric converter filters to HCoV-229E at room temperature for 15 min. (b) Log of virus recovered after exposure to catalytic polymeric filters to HCoV-229E at room temperature for 60 min. The detection limit of the technique is 5 PFU/mL. *N* = 4; error bars correspond to the standard deviation (±SD). Paired student test analysis was used to compare experimental treatments with control: *p-value <0.05, **p-value < 0.01 and ****p-value <0.0001.

## 4 Conclusions

In this work, a CuI antiviral coating has been deposited into a commercial polymeric filter by spray-coating and by dip-coating. Spray-coating methodology was optimized through the tuning of the compressed air pressure and the airbrush-filter distance. Medium distances and pressures (6 cm and 1 bar) were detected as the best conditions to get the best homogeneity, adherence, and biocidal performance. In addition, higher homogeneity, better adherence, and an improved biocidal activity were obtained in the optimized spray-coated filters compared with dip-coated textiles. Consequently, this work introduces the spray-coating methodology as a low cost and simple process to prepare catalytic polymeric filters with biocidal potential for future implementation on a larger scale.

In future stages of this work, the performance and applicability of these filters will be developed, including the biocidal assays against other viral and bacterial pathogens; the enhancement of the filter substrate using alternative polymeric materials such as nanofiber-based filters to improve the functionalization potential; and assays in an aerosol chamber with a bacteriophage φ-29 to simulate real conditions of an airborne transmission.
